# Effects of US7 and UL56 on Cell-to-Cell Spread of Human Herpes Simplex Virus 1

**DOI:** 10.3390/v15112256

**Published:** 2023-11-14

**Authors:** Jun Wang, Ke Wu, Longquan Ni, Chenxuan Li, Ruoyan Peng, Yi Li, Zhaojun Fan, Feifei Yin, Fei Deng, Shu Shen, Xiaoli Wu

**Affiliations:** 1 Key Laboratory of Virology and Biosafety, Chinese Academy of Sciences, Wuhan 430071, China; wangjun@wh.iov.cn (J.W.); ashcan14@163.com (K.W.); near986@163.com (L.N.); lichenxuan22@mails.ucas.ac.cn (C.L.); roy@wh.iov.cn (Z.F.); df@wh.iov.cn (F.D.); 2 University of Chinese Academy of Sciences, Beijing 101499, China; 3 Key Laboratory of Tropical Translational Medicine of Ministry of Education, Hainan Medical University, Haikou 571199, China; pry0302@163.com (R.P.); yinfeifeiff@163.com (F.Y.); 4 The University of Hong Kong Joint Laboratory of Tropical Infectious Diseases, Hainan Medical University, Haikou 571199, China; 5 CAS Key Laboratory of Regenerative Biology, Guangzhou Institutes of Biomedicine and Health, Chinese Academy and Sciences, Guangzhou 510530, China; li_yi1@gibh.ac.cn

**Keywords:** herpes simplex virus 1, cell-to-cell transmission, membrane proteins

## Abstract

Human herpes simplex virus (HSV), a double-stranded DNA virus belonging to the *Herpesviridae* family and alpha herpesvirus subfamily, is one of the most epidemic pathogens in the population. Cell-to-cell spread is a special intercellular transmission mechanism of HSV that indicates the virulence of this virus. Through numerous studies on mutant HSV strains, many viral and host proteins involved in this process have been identified; however, the mechanisms remain poorly understood. Here, we evaluated the effect of the membrane protein genes *US7* and *UL56* on cell-to-cell spread in vitro between two HSV-1 (HB94 and HN19) strains using a plaque assay, syncytium formation assay, and the CRISPR/Cas9 technique. *US7* knockout resulted in the inhibition of viral cell-to-cell spread; additionally, glycoprotein I (*US7*) of the HB94 strain was found to promote cell-to-cell spread compared to that of the HN19 strain. *UL56* knockout did not affect plaque size and syncytium formation; however, the gene product of *UL56* from the HN19 strain inhibited plaque formation and membrane infusion. This study presents preliminary evidence of the functions of *US7* and *UL56* in the cell-to-cell spread of HSV-1, which will provide important clues to reveal the mechanisms of cell-to-cell spread, and contributes to the clinical drugs development.

## 1. Introduction

The human herpes simplex virus 1 (HSV-1; family *Herpesviridae,* genus *Simplexvirus,* species *Human herpesvirus* 1) is mainly transmitted through contact with the skin and mucous membranes. HSV infections are common worldwide, particularly in developing countries [1,2]. It is estimated that 45–90% of the global human population is carrying HSV-1 [3]. Clinical lesions are mostly found in the mouth and lips, resulting in orofacial herpes. The virus may then establish a latent infection in the trigeminal or sacral ganglia, keeping gene expression at a silent level to escape the immune system of the host [1]. Because of this infection-latency-recurrence mechanism, people with HSV-1 infection may not even know that they have been infected and unknowingly spread HSV to others [1].

HSV-1 is a large double-stranded DNA virus whose genome contains 75 open reading frames, and is approximately 152 kb in length. The HSV-1 genome is divided into unique long (U_L_) and short (U_S_) structures that are separated and entrapped by inverted repeats [4]. Gene editing techniques such as CRISPR/Cas9 technology and the BAC scaffold platform are often applied in HSV-1 genome research [5,6]. Owing to the advantages of a large genome, a high proportion of nonessential genes, and a wide infection range, HSV-1 is considered an ideal vector for gene and tumor therapy [7]. HSV-1 virus particles can be divided into three layers, namely, the envelope, tegument, and nucleocapsid. The envelope consists of a phospholipid bilayer and membrane proteins. Envelope proteins play crucial roles in viral entry, intercellular spread, and immune evasion [8]. Phylogenetic analysis of HSV-1 strains based on single-gene or whole-genome sequencing has shown that the variants can be divided into at least three major clades associated with their geographic distribution [9,10].

Members of *Herpesviridae* have evolved a unique mode of cell-to-cell transmission, which is indispensable for the establishment of latent and late recurrent infections after primary infection [11]. Viral particles can directly infect adjacent cells through cell junctions, thus effectively escaping host neutralizing antibodies and other components of the immune system [12]. HSV-1 thus spreads from infected cells to adjacent healthy cells in a cell-free or cell-to-cell manner. A cell-to-cell-transmission-deficient HSV strain could not infect nerve cells in a mouse model to establish a latent infection [13,14], suggesting the importance of cell-to-cell transmission for viral spread in vivo. The gB, gD, gH/gL, gE/gI, gK, and gM proteins have been identified to participate in the cell-to-cell transmission process [15]; however, the molecular mechanisms underlying this process remain unclear.

In this study, two HSV-1 strains (HN19 and HB94) were isolated. Differences in evolutionary status, growth properties, and cell-to-cell spreading abilities were then compared. Furthermore, the CRISPR/Cas9 technology was used to delete or restore the *UL56* and *US7* genes to verify their effects on cell-to-cell transmission. Our findings lay the foundation for analyzing the cell-to-cell propagation mechanism in HSV-1.

## 2. Materials and Methods

### 2.1. Viruses and Cell Lines

HSV-1 HB94, which was isolated in the 1990s from Hubei Province, was deposited at the National Virus Resource Center (NVRC, accession number: IVCAS 6.0180).

African green monkey kidney (Vero) cells from American Type Culture Collection (ATCC, CCL-81, Lot^#^: 60150897), human lung cancer (A549) cells (NVRC, accession number: IVCAS 9.096), and human embryonic kidney (HEK293T) cells (ATCC, CRL-11268, Lot^#^: 62296864) were maintained in Dulbecco’s modified Eagle’s medium (DMEM; Sigma, St. Louis, MO, USA) containing 10% fetal bovine serum (FBS, Gibco, Grand Island, NY, USA) with 5% CO_2_ in an incubator at 37 °C. HSV-1 was proliferated in Vero cells, and virus titer was determined by plaque assays on Vero cells, as previously described [16].

### 2.2. Plasmids and Antibodies

The single guide (sg)RNAs of the HB94 *US7* and *UL56* genes were designed using CRISPOR.org (http://crispor.tefor.net/crispor.py accessed on 1 July 2022) and cloned into pX459, as described previously [16]. The homologous donor repair (HDR) plasmids US7KO/KI and UL56KO/KI were cloned into the pTOPO-blunt vector. The eukaryotic expression system of *US7* and *UL56* genes of the HB94 and HN19 strains were cloned into the pCAGGS vector fused with an S-tag at their C-terminals.

Anti-His antibody (Abcam, Shanghai, China) and β-actin monoclonal antibody (ABclonal, Wuhan, China) were used to verify protein expression or served as an internal control. An anti-ZO-1 polyclonal antibody (Beyotime, Shanghai, China) was used to detect syncytia. Goat anti-Rabbit/Mouse IgG H&L conjugated with horseradish peroxidase (Proteintech, Wuhan, China) and Goat anti-Rabbit IgG H&L (Alexa Fluor ^®^555) (Abcam, Wuhan, China) were used as secondary antibodies according to the manufacturer’s instructions. The cell nuclei were stained with Hoechst 33258 (Beyotime, Shanghai, China).

### 2.3. Sample Collection and Virus Isolation

One human throat swab was collected from a febrile patient with a cough in Hainan province in 2019 (HN19). The swab was diluted in DMEM and centrifuged to remove cell debris. Clarified supernatant (1 mL) was incubated with Vero cells (1 × 10^5^/well) at 37 °C for 1 h and replaced with fresh medium. Cells were maintained at 37 °C for 3–4 days, and blind passages were performed until cytopathic effect (CPE) was observed from almost all cells. Culture supernatants were harvested for RNA sequencing (RNA-seq) to identify viral isolates.

### 2.4. Next Generation Sequencing (NGS)

The nucleic acids of culture supernatants were extracted using a PureLink^TM^Viral RNA/DNA Mini Kit (Invitrogen™, Carlsbad, CA, USA, Lot^#^:12280050), and then NGS (RNA-seq) carried out using the Illumina Hiseq 3000 platform according to the manufacturer’s instructions (Illumina, San Diego, CA, USA). The sequencing data were subjected to quality control (FastQC v0.11.9; Trimmomatic v0.39). The clean reads were assembled according to the VirGA pipeline (https://virga.readthedocs.io/en/latest/, accessed on 20 June 2022), and HSV-1 strain 17 (JN555585.1) was used as the reference genome. BLASTn and BLASTx comparisons were performed to identify virus-related sequences and annotate *HSV-1* genes.

### 2.5. Construction of Recombinant Virus

Six-well plates seeded with HEK293T cells (30–40% confluence) were transfected with a mixture containing the sgRNA1 (1 µg), sgRNA1/2 (1 µg), and HNR DNA plasmids (2 µg) using calcium phosphate (Beyotime, Shanghai, China). The cells were incubated with the mixture at 37 °C for 8 h, and were gently shaken to resuspend the calcium phosphate precipitation. Then, the supernatants were removed and replaced with fresh medium containing 10% FBS and 100 mM SCR7 (diluted with DMSO to a final concentration of 100 µm, MedChemExpress, Monmouth Junction, NJ, USA). At 24 h post-transfection (h.p.i.), the cells were incubated with HSV-1 (strain HB94) at a multiplicity of infection (MOI) to generate the recombinant viruses HB94-US7KO and HB94-UL56KO, which contained the genome with *US7* and *UL56* gene knockouts, respectively. The recombinant viruses HB94-HN19US7KI and HB94-HN19UL56KI, which had *US7* and *UL56* gene knock-ins, respectively, were constructed based on HB94-US7KO and HB94-UL56KO. After the cells were incubated with virus for 1 to 2 h at 37 °C, the cells were gently washed with DMEM, and fresh DMEM with 2% FBS and 100 mM SCR7 was added. The cells and supernatant were collected at 24 h.p.i., frozen and thawed three times at −80/37 °C, and the supernatant collected by centrifugation to obtain the P0 recombinant virus.

### 2.6. Recovery of Plaque Phenotype under Exogenous Expression

Vero cells (1 × 10^5^/well) were transfected with 2 μg of plasmids pUS7-HN19, pUS7-HB94, pUL56-HN19, or pUL56-HB94, expressing US7 or UL56 from the HN19 or HB94 strain, using lipofectamine 3000 (Invitrogen™, Carlsbad, CA, USA). The expression of US7 and UL56 in cells was detected using Western blotting, as previously described [16]. Subsequently, US7- or UL56-expressing cells were infected with 50 PFU of the HB94-US7KO or HB94-UL56KO strains. Plaque formation induced by HSV-1 infection was characterized as previously described [16]. Images were captured using an EVOS FL autoinverted microscope (Thermo Fisher, Carlsbad, CA, USA). The size of the plaques was measured using the built-in software (Rev 26059). Significant difference was analyzed using SPSS (version 19.0.0).

### 2.7. Syncytial Formation Assays

Syncytia formation induced by HSV-1 infection was investigated using a previously described method with slight modifications [17]. Vero cells (1 × 10^5^ per well) seeded in 12-well plates were incubated with HSV-1 (MOI = 0.01) at 37 °C for 1 h. Then, the supernatants were removed and the cells washed three times with DMEM, and cells were maintained in DMEM containing 2% FBS. At 24 h.p.i., cells were fixed with 4% paraformaldehyde and permeabilized with 0.2% Triton X-100 (Sigma-aldrich, St. Louis, MO, USA, Lot^#^:WXBD4860V). The formation of syncytia was visualized using an immunofluorescence assay (IFA), which immunostained the ZO-1 protein to display the cell junction. Cell nuclei were stained with Hoechst 33258 (Beyotime, Shanghai, China, Lot^#^:C1011), and images were captured by an EVOS FL autoinverted microscope. Cell numbers were counted using ImageJ software (v1.51s). Cell foci containing fewer than three nuclei are thought to form syncytia [17].

### 2.8. Bioinformatic Analysis

Sequence alignment was performed using ClustalW with the two isolated HSV-1 strains (HB94 and HN19), and 63 HSV-1 sequences were deposited in GenBank. A phylogenetic tree was constructed using the Maximum Likelihood method with bootstrap values of 1000 for the UL and US regions using MEGA7. The HSV-1-related reads identified by RNA-seq were mapped to the viral genome sequence using bowtie2 and displayed using IGV (v2.3). The alignment of *US7* and *UL56* was shown using GeneDoc 2.7, and the secondary structures of *UL56* and *US7* were predicted using Psipred tools (v4.0) (http://bioinf.cs.ucl.ac.uk/psipred/&uuid=a895f73e-e260-11ec-ab91-00163e100d53, accessed on 1 June 2022).

### 2.9. GD Copy Number Statistics in Genome Replication Stage

Vero cells (3 × 10^5^ cells/well) seeded in 12-well plates were inoculated with HSV-1 (MOI = 5) at 37 °C for 1 h. The virus was removed and the cells were washed three times with DMEM. Then, the cells were inoculated with 1 mL 2% FBS DMEM at 37 °C. Supernatants and cells were collected at 2, 6, 12, and 24 h.p.i. Supernatants (200 μL) were harvested for viral nucleic acid extraction using a PureLink^TM^ Viral RNA/DNA Mini Kit (Thermo Scientific, MA, USA). The gD sequence of HB94 strain was used to construct the DNA standard, and the samples at different time points were quantitatively detected using qPCR, using gD-QF/QR (gD-QF:5′-ACGACTGGACGGAGATTACA-3′, gD-QR:5′- GGAGGGCGTACTTACAGGAG-3′) primers. Quantitative PCR was performed using SYBR^®^ qPCR Mix (Takara, Osaka, Japan) with primers as previously described [16]. Each experiment was performed in triplicate.

### 2.10. One-Step Growth Curve Analyses

Vero cells (3 × 10^5^ cells/well) were seeded in 12-well plates and infected with HSV-1 (MOI = 0.01 or 5). Supernatants and cells were harvested from each sample at the indicated time points and subjected to three freeze–thaw cycles. Viral titers were determined in Vero cells using a plaque assay. Each experiment was performed in triplicate. The growth curve was plotted using GraphPad Prism 8.0 software (GraphPad Software Inc.).

### 2.11. Comparative Experiment on the Dynamics of Cell Invasion Stage

Vero cells were seeded at a density of 3 × 10^5^ cells per well in a 12-well plate and cultured overnight. Prior to viral infection, the growth medium was replaced with prechilled medium and placed at 4 °C for 30 min. Subsequently, each well was infected with 120 PFU HSV-1 (HB94 or HN19), allowing adsorption at 4 °C for 1 h. Then, the viral inoculum was removed, and the plate was washed three times with serum-free DMEM at room temperature. Finally, 0.5 mL of DMEM was added to each well, and the plate was placed in a 37 °C incubator to enable the viral entry process. At 0, 10, 20, 30, 45, and 60 min postincubation at 37 °C, the respective well’s medium was aspirated and treated with a low-pH citrate buffer for 2 min to inactivate nonentered viral particles. The citrate buffer was removed, and the cells were washed twice with serum-free DMEM. Subsequently, DMEM was added back, and the plate was returned to the incubator for a total incubation time of 60 min at 37 °C. Finally, the culture medium was removed, and a layer of agar overlay solution was added. After solidification, the plate was placed back in the incubator for 3 days. The control group did not undergo treatment with the low-pH citrate solution. After staining with crystal violet fixing solution, the number of plaques in each well was counted, and the ratio of plaque number at different time points to that in the control group was calculated. The results came from three independent replications.

### 2.12. Relative mRNA Expression of US7 and UL56

Vero cells (3 × 10^5^ cells/well) were infected 1 MOI HSV-1 (HB94, HN19, HB94-US7KO, HB94-HN19US7KI or HB94UL56KO), and the cell were collected at 48 h.p.i. RNA was extracted using the TaKaRa MiniBEST Viral RNA/DNA Extraction Kit Ver.5.0 (TaKaRa, Code No.9766, Japan). DNA removal and reverse transcription experiment was completed by PrimeScript™ RT reagent Kit with gDNA Eraser (TaKaRa, Code No.RR047A, Japan). For internal reference genes, β-Actin was chosen as the reference gene for the cells, and HSV-gD was selected as the reference gene for the virus. US7 and UL56 mRNA was detected by TB Green Premix Ex Taq II (Tli RNaseH Plus) (TaKaRa, Code No.RR820A/B, Japan) for real-time PCR reactions. Data analysis and graphing were performed using GraphPad Prism 8.

## 3. Results

### 3.1. Stronger Cell-to-Cell Transmission Ability of HB94 Than that of HN19

**Figure 1:**
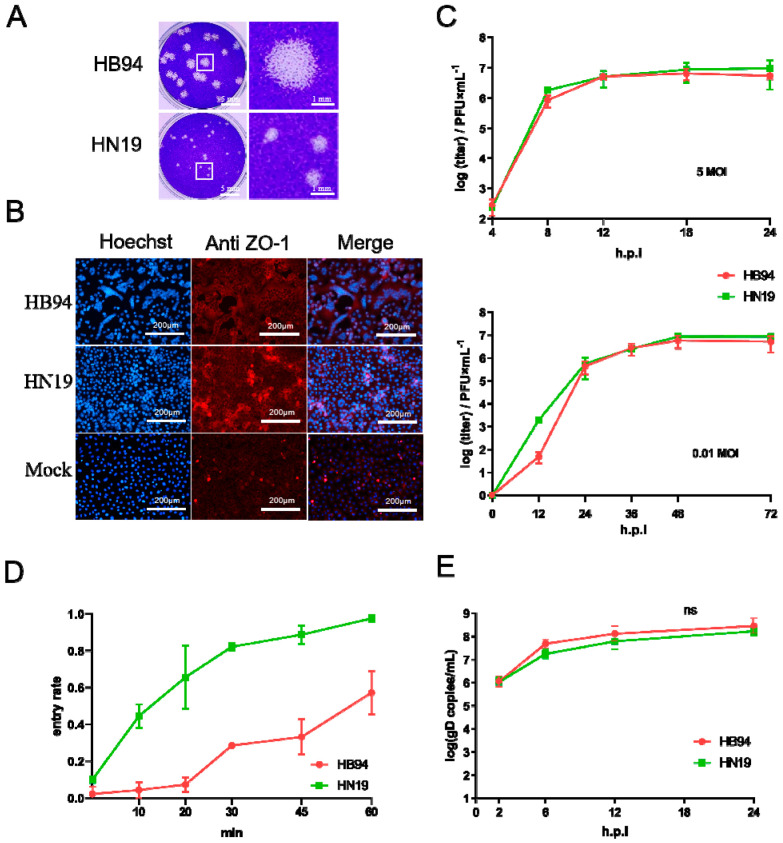
Phenotypic difference between HB94 and HN19. (**A**) Difference in syncytial size. Vero cells (3 × 10^5^ cells/well) were infected by 50 PFU HB94 or HN19 strains and cultured for 3 days. The white squares shows the enlarged plaque image (right) with a bar of 1 mm. (**B**) Difference in syncytia size. Vero cells were infected by HSV-1 at 0.01 MOI cultured for 24 h. The ZO−1 protein indicates the ability of syncytia. (**C**) One-step growth curve at 5 or 0.01 MOI. The cellular supernatants were harvested at 12, 24, 36, 48, and 72 h.p.i., and the viral titers were detected by plaque assay. (**D**) Growth curve of the ratio of virions entering cells. Vero cells (3 × 10^5^ cells/well) were infected by 120 PFU HB94 or HN19 strains at 4 °C for 1 h. Virus supernatant was replaced by DMEM medium and cultured at 37 °C for viral entry. Then, the medium was replaced by low-PH citrate buffer solution at 0, 10, 20, 30, 45, and 60 min. Virions that did not enter the cell were inactivated by citric acid buffer solution. After that, the number of plaques in the experimental group and the control group was calculated to determine the proportion of virus entry. (**E**) Growth curve of *gD* gene copies at 5 MOI. The cellular supernatants were harvested at 2, 6, 12, 18, and 24 h.p.i., and the viral gD gene copies were detected by qPCR. Ns: not significant.

Two HSV-1 strains (HB94 and HN19) were isolated from human samples (Table 1), and their growth characteristics were compared in Vero cells. The HSV-1 strains HB94 and HN19 showed different plaque-forming abilities. HB94 induced the formation of more plaques of larger size than HN19. The average plaque size produced by HB94 was 3.76 mm^2^, whereas the mean size for HN19 was 0.53 mm^2^ (Figure 1A). This suggests that HSV-1 HB94 may have a higher cell-to-cell transmission ability than HN19. ZO-1 is a cytoskeletal protein in the tight junction structure of cells that shows the structure of the plasma membrane and the range of the cytoplasm using IFA [18]. Growth property analyses showed that both HB94 and HN19 strains produced CPE at 24 h after infection with 0.01 MOI (Figure 1B). However, the HB94 strain strongly induced fusion of the plasma membrane between cells, and the nuclei closely gathered on the same plane to form obvious multinucleated cells, namely syncytia (Figure 1B). In the HN19 group, CPE showed greater detachment of cells, stacking of cell clusters in the lesion area, a relatively independent cytoplasm of cells, and only a small portion of membrane fusion (Figure 1B). By measuring the ratio of the syncytial area to the total nuclear area, we found a significant difference in the ratio of the syncytial area between the HB94 and HN19 strains (Figure S1A). Through plaque and syncytial formation tests, we found that the plaque area and syncytial size of the HB94 strain after infection were significantly larger than those of the HN19 strain, indicating that the HB94 strain had stronger cell-to-cell transmission ability than the HN19 strain.

We further compared the differences between the two strains at three stages: cell invasion, genome replication, and progeny virus amplification. The one-step growth curve showed no significant difference in growth characteristics between the HB94 and HN19 strains with 0.1 or 5 MOI (Figure 1C). After incubation for 60 min, the proportion of invasion reached 95% (Figure 1D). However, the invasion rate of HB94 was slower (Figure 1D). After incubation for 60 min, the invasion rate was approximately 50%, and it took 2 h for the viral particles to invade (Figure S1B). There was no significant difference between HB94 and HN19 in the number of genome copies during the amplification period, indicating that the HB94 and HN19 strains have the same ability for genome replication and synthesis and assembly of progeny viruses (Figure 1E).

In conclusion, HB94 showed a stronger cell-to-cell transmission ability than HN19 in plaques and syncytia, which was not caused by differences in invasion, replication, and amplification.

### 3.2. US7 and UL56 Genes Showed Differences in HB94 and HN19 Strains via Phylogeny and Identity Analyses

**Figure 2:**
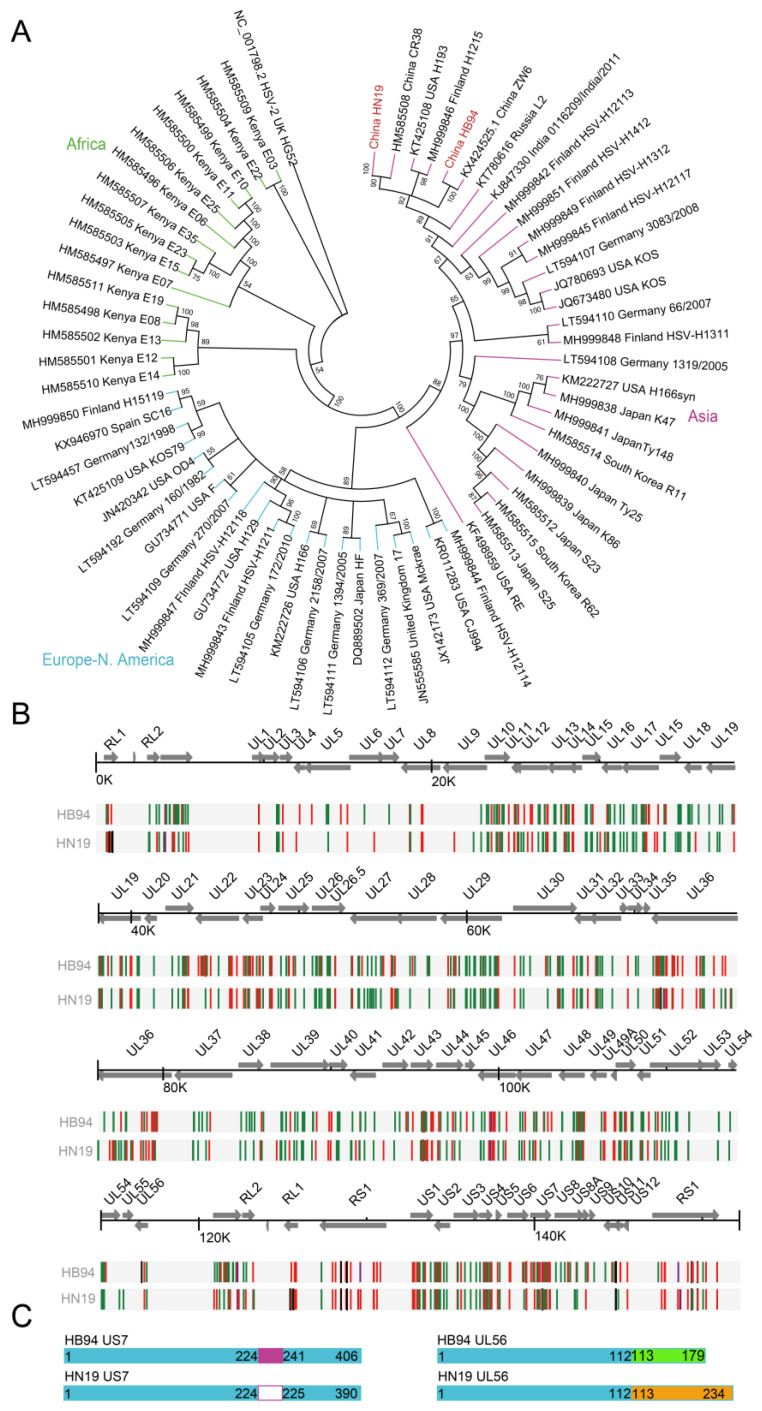
Analysis of genetic evolution and genome differences of HB94 and HN19. (**A**) Phylogenetic evolutionary tree. The blue branch represents the Europe-N. America clade, the green branch represents the Africa clade, and the purple branch represents the Asia clade. The HB94 and HN19 strains are marked in red. The (**B**) Gene annotation and mutation diagrams; black, synonymous mutation; green, nonsynonymous mutation; red, insert. ORFs are indicated by arrows. The direction of the arrows indicates the orientation of gene transcription. (**C**) *US7* and *UL56* gene schematic of HB94 and HN19. The right side represents differences in the US7 gene, and the left side represents differences in the UL56 gene.

To better understand the differences in the cell-to-cell transmission abilities between the two strains, we compared their genome sequences and characterized their evolutionary relationships with those of other HSV-1 strains. As previously described [9,10], the HSV-1 strains were divided into three clades based on their geographical regions: African, Asian, and European and North American groups (Figure 2A). The HB94 and HN19 strains belong to the Asian clade and were closely related to two strains from China (CR38 and ZW6). Both strains shared high nucleotide (99.1%) and protein (98.9%) sequence similarity (Table 2).

The genomic sequences of HB94 and HN19 had 631 and 575 single nucleotide polymorphisms (SNPs), of which 260 and 234 SNPs were nonsynonymous with the reference strain 17 (JN555585.1) (Figure 2B), respectively. Moreover, there were 45 encoded proteins with mutations in HB94 and HN19, and we classified the protein sequences into envelope/membrane-related proteins, replication/regulatory-related proteins, tegument proteins, capsid proteins, nuclear egress-related proteins, encapsidation-related proteins, and unknown function proteins according to function (Figure S2). We found that the differences in membrane proteins and replication/regulatory proteins were larger than those in other proteins (Figure S2). *US7*, *UL56*, and *US11* accounted for more than 5% of the total protein length (Figure S2). The matrix protein *US11* is a multifunctional regulator with RNA-binding ability that can inhibit host antiviral immunity [19,20,21]. Membrane proteins play key roles in HSV-1 transmission [22]. Of the analyzed membrane proteins, the *US7* and *UL56* genes exhibited high diversity between HB94 and HN19 (Figure S2). Gene annotation and sequence comparison with other Chinese strains showed that HB94 has a frame shift mutation because of a two-nucleotide deletion (334–335, CT) in the *UL56* gene, and the *US7* gene has an additional sequence of 16 amino acids (225–240) (Figure 2C and Figure S3). Since *US7* and *UL56* can regulate the transport of progeny viruses, as previously described [23,24,25], the differences in *US7* and *UL56* of HSV-1 HB94 may indicate that they function differently from other strains, and thus affect cell-to-cell transmission and the phenotype of infection in cells. 

### 3.3. US7 Promoted the Formation of Plaques, and UL56 of HN19 Inhibited the Formation of Plaques

**Figure 3:**
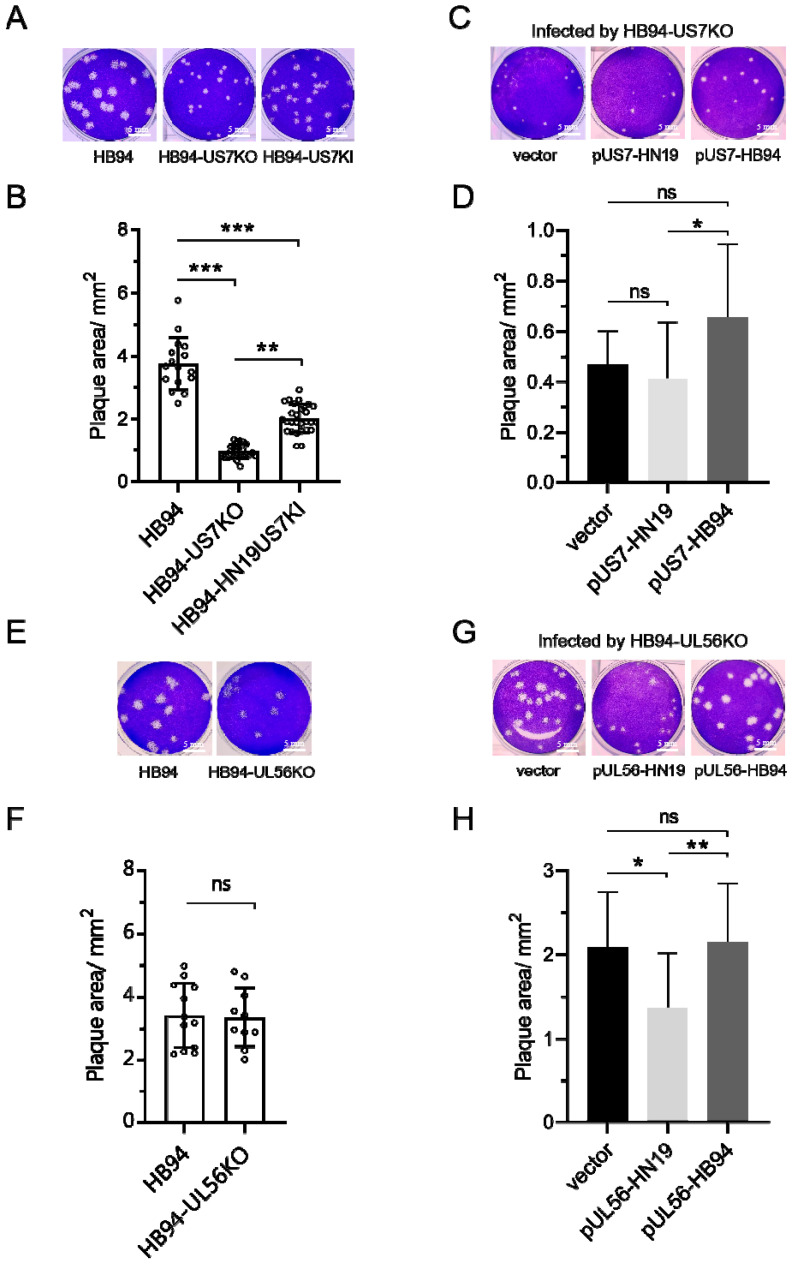
Effects of *US7* and *UL56* on HSV-1 plaque formation. (**A**,**B**) Analysis of plaque size and area of HB94 strain by deletion and restoration of *US7* gene. Vero cells (3 × 10^5^ cells/well) were infected by 50 PFU HB94, HB94-US7KO, and HB94-HN19US7KI strains and cultured for 3 days. (**C**,**D**) Analysis of plaque size and area of exogenous *US7* gene expression in HB94-US7KO strain. The eukaryotic expression plasmid pUS7-HN19 or pUS7-HB94 was transfected into Vero cells (1 × 10^5^ cells/well) and infected with 50 PFU HB94-US7KO after 24 h. (**E**,**F**) Analysis of plaque size and area of HB94 strain with deletion of *UL56* gene. Vero cells (3 × 10^5^ cells/well) were infected by 50 PFU HB94 and HB94-UL56KO strains and cultured for 3 days. (**G**,**H**) Analysis of plaque size and area of exogenous *UL56* gene expression in the HB94-UL56KO strain. The eukaryotic expression plasmid pUl56-HN19 or pUl56-HB94 was transfected into Vero cells (1 × 10^5^ cells/well) and infected with 50 PFU HB94-Ul56KO after 24 h. Similar data were obtained from at least two independent experiments. Stats: one-way ANOVA with a Tukey multiple comparisons; * *p* < 0.05, ** *p* < 0.01, *** *p* < 0.001. ns: not significant.

**Figure 4:**
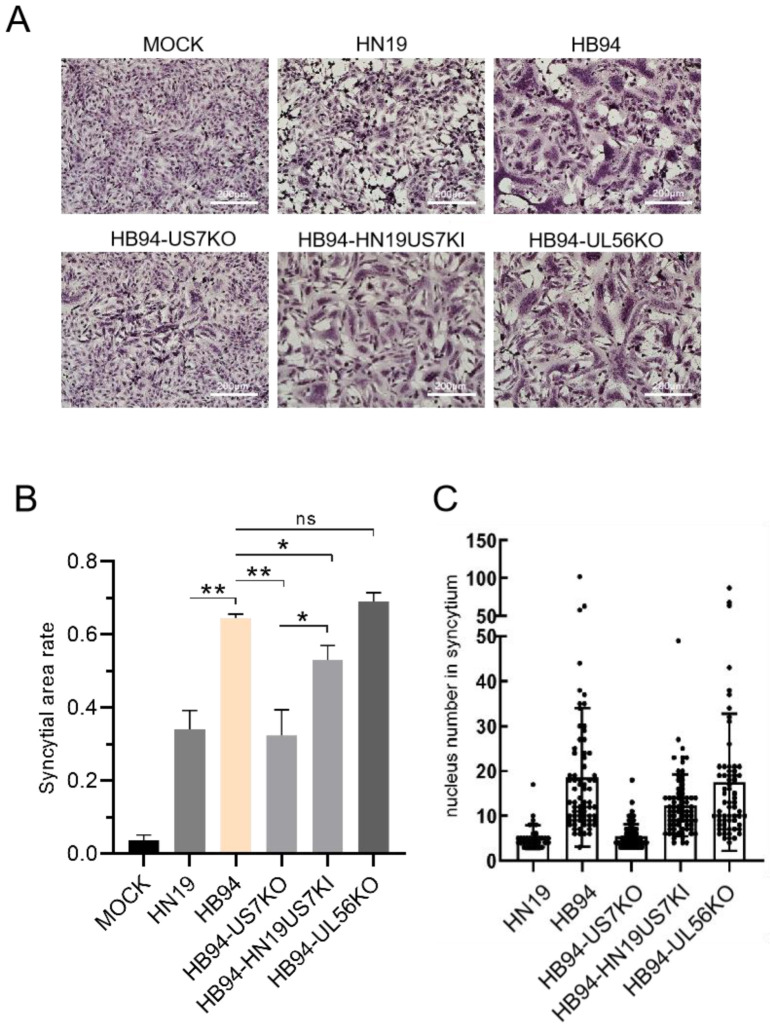
Effects of *US7* and *UL56* on HSV-1 syncytia. (**A**) Effects of HB94 strain *US7* or *UL56* genes on deletion and restoration of plaques. Vero cells (3 × 10^5^ cells/well) were infected with 0.01 MOI HB94 or HN19 strains and cultured for 3 days. Bar, 200 μm. (**B**) Syncytial ratio. Stats: one-way ANOVA with a Tukey multiple comparisons; * *p* < 0.05, ** *p* < 0.01. (**C**) Number of nuclei in a single syncytium. ns: not significant.

The size of plaques formed by HB94-US7KO were significantly smaller than that formed by wild-type HB94 (*p* < 0.001), indicating that *US7* can promote plaque formation (Figure 3A,B). We then constructed the HB94-HN19US7KI strain by replacing the *US7* of HB94 with HN19 *US7,* and measured the plaque size. *US7* from HN19 restored plaque formation of HB94-US7KO in Vero cells (Figure 4A); however, the mean size was still less than that of the wild-type HB94 strain (*p* < 0.001, Figure 4B). Expression of the *US7* and *UL56* proteins of HN19 and HB94 was confirmed by Western blotting in cells transfected with the protein expression plasmids (Figure S3A,B). Plaque assays showed that *US7* of strain HN19 could not rescue the *US7* knockout HB94 virus to form as many plaques as the US7 of strain HB94 (Figure 3C,D). The mRNA level of US7 gene in HN19 and HB94 was compared, and the US7 gene level in HN19 was significantly lower than that in HB94 (Figure S5A). These results suggest that *US7* can improve cell-to-cell transmission, and that *US7* from HB94 can promote the formation of plaques more effectively than *US7* from HN19.

Because we failed to harvest the HB94-HN19UL56KI strain, we only compared the plaque size of the HB94-UL56KO strain with that of the wild-type. The sizes of the plaques formed by HB94-UL56KO and wild-type HB94 were similar, indicating that *UL56* of HB94 did not participate in cell-to-cell transmission (Figure 3E,F). The *UL56* of strain HN19 inhibited plaque formation, but not when the *UL56* of HB94 was present; the plaque area of the HB94-UL56KO strain was smaller than that of the control group transfected with an empty vector (*p* < 0.05, Figure 3G,H), indicating that the *UL56* gene product of the HN19 strain can inhibit the formation of plaques. These partial results suggest that *US7* can promote plaque formation, and that *UL56* may participate in cell-to-cell transmission.

### 3.4. US7 Promoted Syncytial Formation, and UL56 Had no Effect on Syncytial Formation 

Syncytia formation assays were performed to evaluate the effect of *US7* and *UL56* on membrane fusion. We confirmed the difference in syncytial formation between HN19 and HB94 by staining (Figure 4A), and the proportion of syncytial formation in HB94 was significantly higher than that in HN19 (Figure 4B). The effects of *US7* and *UL56* deletions on syncytia formation were further evaluated. After the deletion of *US7*, the proportion of syncytial formation was significantly reduced, and *US7* in HN19 partially restored syncytial formation, suggesting that *US7* promotes syncytial formation. In contrast, when *UL56* was deleted from HB94, syncytium formation was not affected. These results indicated that *US7* promotes membrane fusion and cell invasion.

## 4. Discussion

In this study, the HB94 and HN19 strains were found to have significant differences in phenotypes, such as plaque phagocytosis and syncytium induction, suggesting differences in their cell-to-cell transmission ability. To further explore the reasons for the differences in cell-to-cell transmission ability in HSV-1 isolates, we conducted whole-genome sequencing and completed gene annotation of the HB94 and HN19 strains. At the same time, by comparing *US7* and *UL56* relative mRNA levels of HB94 and HN19 strains, it was found that the *US7* mRNA level of HB94 was significantly higher than that of HN19 strains, and their *UL56* mRNA levels were consistent, which indicates that *US7* may promote cell-to-cell transmission ability. Previous studies have reported that *US7* encodes the viral glycoprotein gl, which forms a heterodimer with gE on the membrane, regulates the transport of progenerating viruses to the intercellular junction, and directly participates in cell-to-cell transmission [25]. This is consistent with the conclusion that *US7* is a key gene involved in the cell-to-cell transmission of HSV-1. We further found that *US7* can promote the formation of viral plaques and syncytia.

*UL56* is a type II membrane protein that is anchored to the C-terminus and localized to both the Golgi apparatus and cytoplasmic vesicles. It is known to be one of the viral neurotoxic factors, and has been shown to interact with host Nedd4 family ubiquitin ligases [23,24,26]. Previous studies have found that *UL56* did not affect the expansion of HSV, but the number of extracellular infectious virions decreased after deletion of the *UL56* protein in HSV-2, suggesting that *UL56* is involved in the transport and release of progeny viruses into the extracellular system, and indirectly affects the intercellular transmission of the virus [24]. In this study, we found that the *UL56* knockout of HB94 had no effect on the formation of plaques and syncytia, but the *UL56* protein product of HN19 inhibited the formation of plaques, suggesting the influence of *UL56* on the cell-to-cell transmission ability of HSV-1. Unfortunately, as we were unable to obtain a recombinant strain of HB94-UL56KI, we could not further verify the effect of *UL56* on cell-to-cell transmission.

Based on the experimental results of this study, we propose a mechanism by which *US7* and *UL56* affect HSV-1 cell-to-cell transmission (Figure S6). When a progeny virus matures in the cytoplasm, it faces two transport directions, extracellular and cell junctions, which correspond to cell-free and cell-to-cell interactions, respectively. The *UL56* protein is located in the TGN (trans-Golgi network) transport system and participates in the release of the progeny virus from the cell. When *UL56* loses its function owing to mutations, the proportion of viruses directed to the outside of the cell decreases, and more virus particles are transferred to the transport system flowing to the cell junction, which enhances cell-to-cell transmission. gI (*US7*) combines with gE to form a functional complex with matrix proteins (*UL11*, *UL16*, and *UL21*) that transport progeny viruses to intercellular junctions and mediate cell-to-cell transmission. Owing to the existence of mutations, the gI of the HB94 strain may have a stronger ability to collect progeny viruses or bind to downstream cell receptors than the HN19 strain, showing stronger cell-to-cell transmission ability.

Because we failed to obtain the *UL56* gene-restoring recombinant virus, we could not further evaluate the effect of the *UL56* gene of the HN19 strain on cell-to-cell transmission. Due to the limited data, we failed to establish how the proteins coordinate during the cell-to-cell transmission process. In the future, we hope to further explore cell-to-cell propagation mechanisms.

## 5. Conclusions

This study investigated two HSV isolates (HB94 and HN19). By measuring the size of the plaques and syncytia produced by infected cells, we confirmed a significant difference in the cell-to-cell transmission ability between the two viruses. Based on the difference between the amino acid sequence and the reported gene function, it was considered that the gene sequence difference between *UL56* and *US7* might be the reason for this difference. We found that *US7* enhances cell-to-cell transmission. Deletion of *UL56* did not affect the formation of viral plaques or the syncytial system; however, its protein products inhibited the formation of viral plaques, suggesting that *UL56* may be involved in regulating cell-to-cell transmission mechanisms. The influence of *US7* and *UL56* on cell-to-cell transmission will help elucidate the transmission mechanism of HSV-1. The key sites and fragments with differences between the *UL56* and *US7* sequences provide clues for further functional studies.

## Figures and Tables

**Table 1:** The information of HSV-1 isolated in this paper.

Sample No.	Strain Code	NVRC Accession Number	Subject	Site	Province	Date
1	HB94	CSTR:16533.06.IVCAS 6.0180	N/A	N/A	Hubei	1994
2	HN19	CSTR:16533.06.IVCAS 6.7531	Patient female 40	Lip	Hainan	2019

N/A: Not applicable.

**Table 2:** HSV-1 identity in China.

Strain Code	No. of Reads Used for Assembly	Avg coverage	Genome Length	Accession No.	Avg ORF Identity with HB94 (nucl/aa)	Avg ORF Identity with HN19 (nucl/aa)	Avg ORF Identity with ZW6 (nucl/aa)	Avg ORF Identity with CR38 (nucl/aa)
HB94 ^a^	46.4 × 10^6^	46278 X	151,002	NAAADWDO10000000	100/100	99.1/98.9	99.3/98.5	99.1/98.3
HN19 ^a^	7.8 × 10^6^	7623 X	152,073	NAAADWEO10000000	99.1/98.9	100/100	99.6/99.5	99.6/99.3
ZW6 ^b^	N/A	N/A	152,090	KX424525	99.3/98.5	99.6/99.5	100/100	99.6/99.2
CR38 ^b^	N/A	N/A	151,427	HM585508	99.1/98.3	99.6/99.3	99.6/99.2	100/100

^a^: The sequence from China National GeneBank DataBase. ^b^: The sequence from GeneBank database. N/A: Not applicable.

## Data Availability

The Sequencing data were deposited in China National GeneBank DataBase. The other data presented in this study are available from the corresponding author upon reasonable request.

## Supplementary Materials

The following supporting information can be downloaded at: https://www.mdpi.com/article/10.3390/v15112256/s1, Figure S1: Phenotypic difference between HB94 and HN19; Figure S2: Variation analysis of protein sequence between HB94 and HN19 strains; Figure S3: The protein sequence alignment of *US7* and *UL56*; Figure S4: The protein expression of *US7* and *UL56* in eukaryotic expression system; Figure S5: The relative mRNA expression of *US7* and *UL56* in different HSV-1. Figure S6: *US7* and *UL56* affect cell-to-cell propagation patterns.
