# Supplementary material for: Effects of US7 and UL56 on Cell-to-Cell Spread of Human Herpes Simplex Virus 1
Source: Viruses. 2023 Nov 14;15(11):2256. doi: 10.3390/v15112256 (PMC10675736; doi:10.3390/v15112256)
Supplement: Supplementary file 1 [file viruses-15-02256-s001.zip › viruses-2680166-supplementary.pdf]

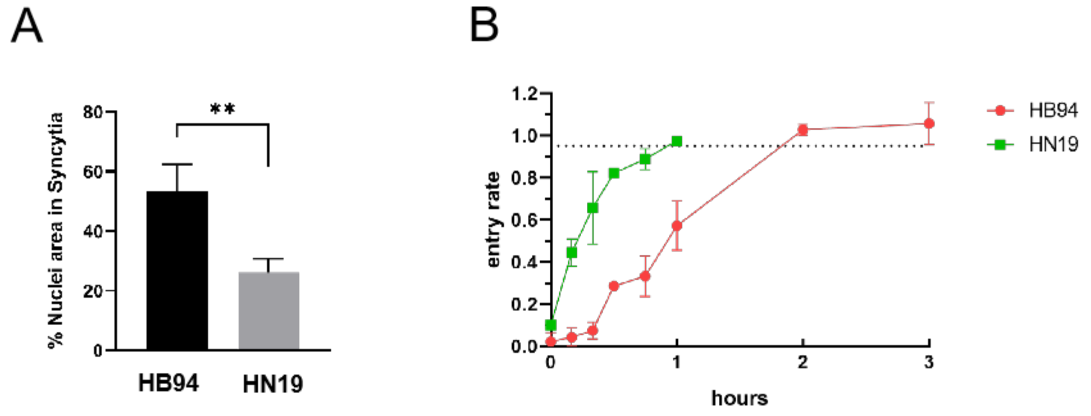

Figure S1. Phenotypic difference between HB94 and HN19. A. Nuclei area in Syncytia. Vero cells (3 × 10^5 cells/well) were infected by 50 PFU HB94 or HN19 strains and cultured for 3 days. Stats: one-way ANOVA with a Tukey multiple comparisons; ** $p < 0.01$. B. Growth curve of the ratio of virions entering cells. Vero cells (3 × 10^5 cells/well) were infected by 120 PFU HB94 at 4 °C for 3 h or HN19 strains at 4 °C for 1 h. Virus supernatant was replaced by DMEM medium and cultured at 37 °C for viral entry. Then, the medium was replaced by low-PH citrate buffer solution at 0, 10, 20, 30, 45, 60 min, 2 h and 3 h. After that, the number of plaques in the experimental group and the control group was calculated to determine the proportion of virus entry.

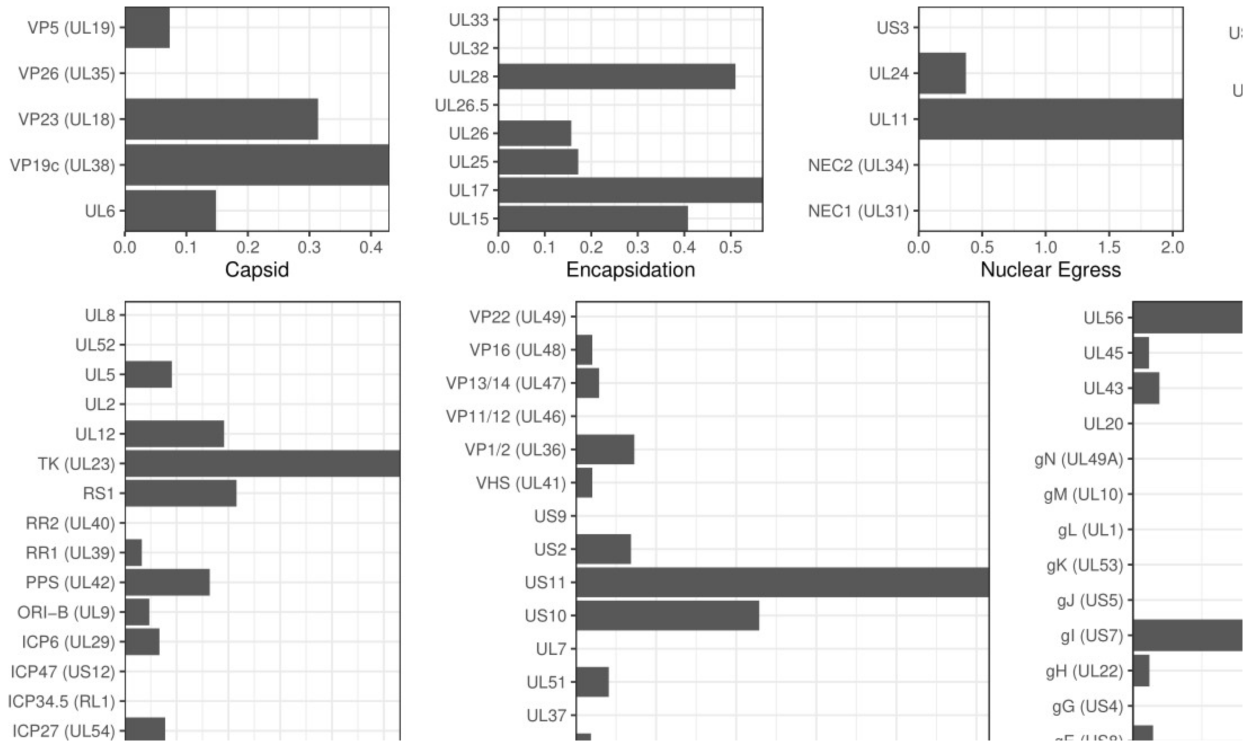

Figure S2. Variation analysis of proteins' sequence between HB94 and HN19 strains. The proteins encoded by the virus were grouped according to their function, and the ratio of amino acid variation to the total length of the protein of the HN19 strain and HB94 strain was shown.

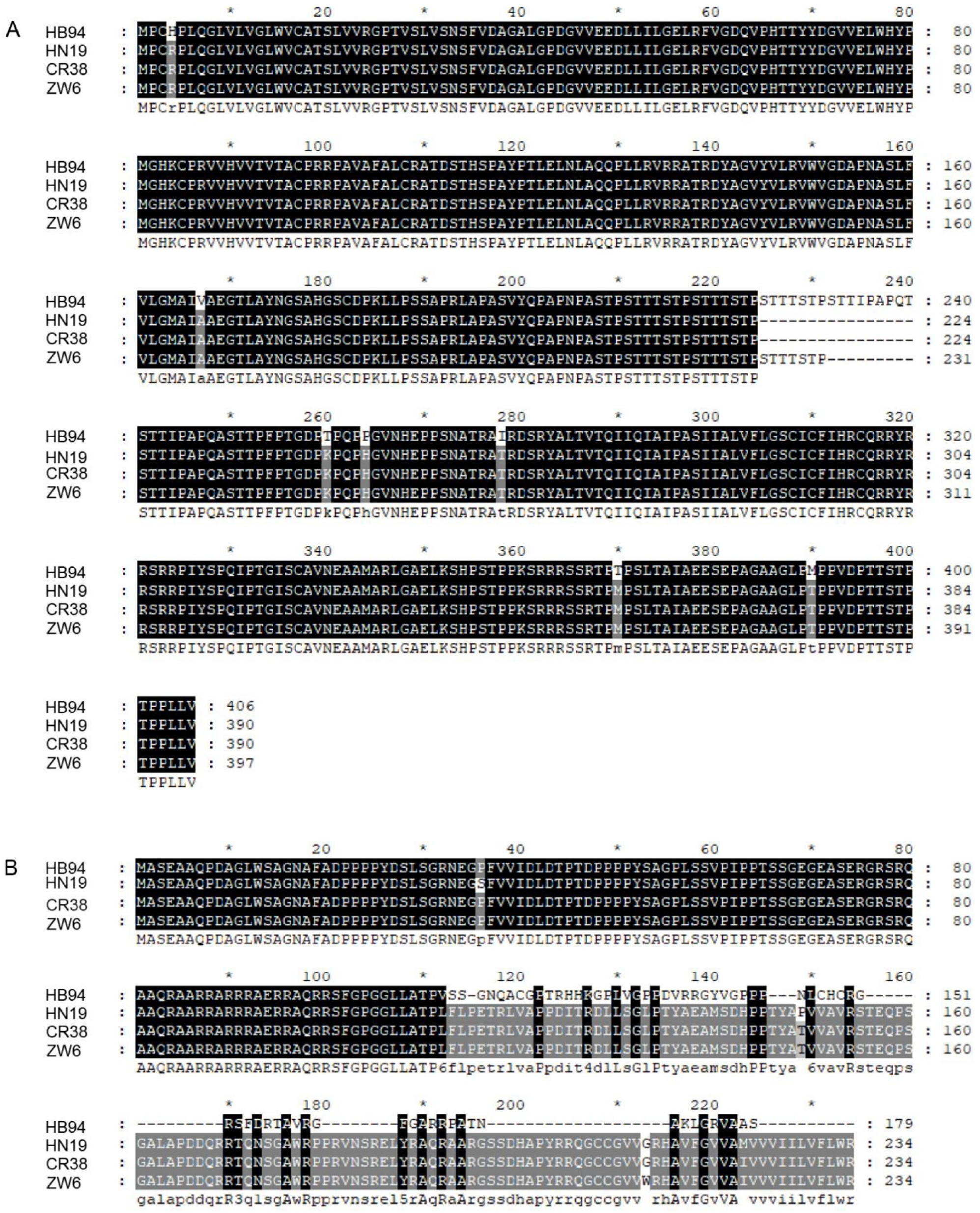

Figure S3. The protein sequence alignments of *US7* and *UL56*. (**A**) The protein sequence alignments of *US7*. (**B**) The protein sequence alignments of *UL56*. The black area represents sequence conservation is 100%, and the gray area indicates conservation is greater than 80%.

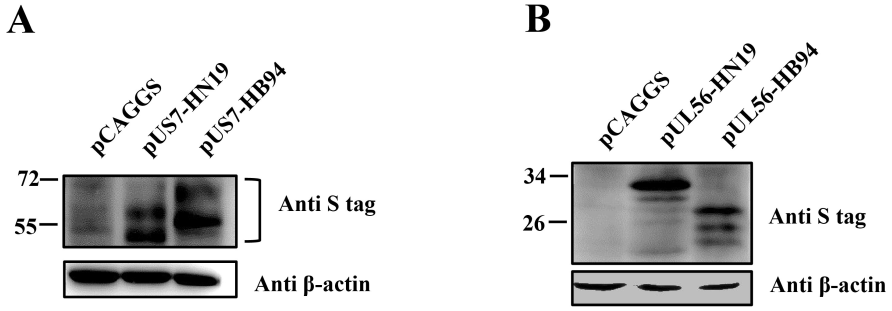

Figure S4. The protein expression of *US7* and *UL56* in eukaryotic expression system. The protein expression of *US7* (**A**) and *UL56* (**B**) was detected by S tag in eukaryotic expression system.

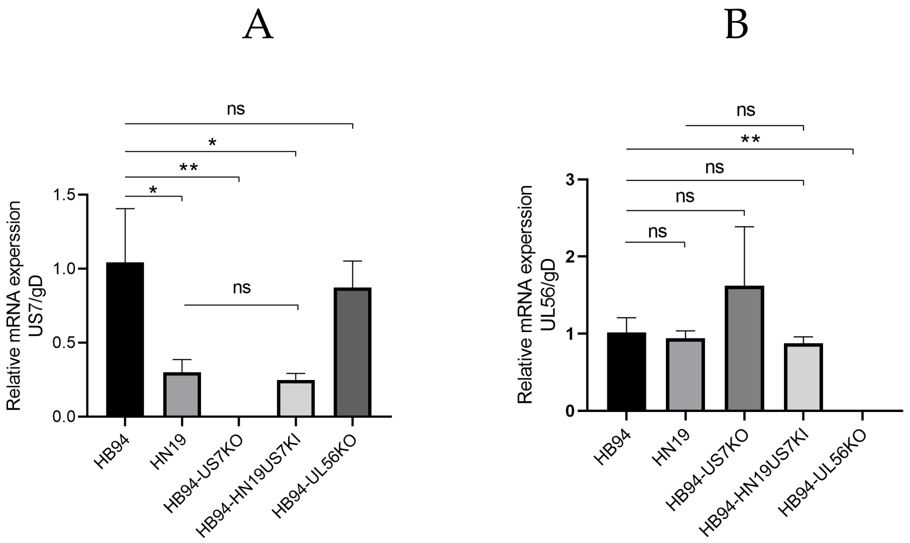

Figure S5. The relative mRNA expression of *US7* and *UL56* in different HSV-1. (**A**) and (**B**) represent mRNA level of US7 and UL56 gene relative to GD gene. Each group of experiments was repeated 3 times, and the statistical method was t-test; *$p < 0.05$, **$p < 0.01$, ns: not significant.

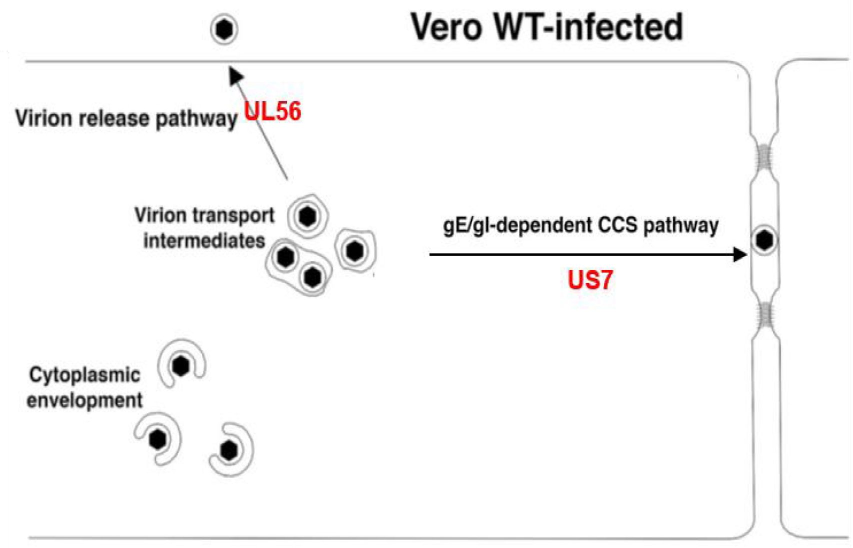

Figure S6. *US7* and *UL56* affect cell-to-cell propagation patterns.
